# High infectivity and unique genomic sequence characteristics of *Cryptosporidium parvum* in China

**DOI:** 10.1371/journal.pntd.0010714

**Published:** 2022-08-22

**Authors:** Ruilian Jia, Wanyi Huang, Ni Huang, Zhengjie Yu, Na Li, Lihua Xiao, Yaoyu Feng, Yaqiong Guo

**Affiliations:** 1 State Key Laboratory of Bioreactor Engineering, School of Resources and Environmental Engineering, East China University of Science and Technology, Shanghai, China; 2 Center for Emerging and Zoonotic Diseases, College of Veterinary Medicine, South China Agricultural University, Guangzhou, Guangdong, China; Universidade Federal de Minas Gerais, BRAZIL

## Abstract

Zoonotic *Cryptosporidium parvum* infections are mainly caused by IIa and IId subtypes. As most biological characterizations have been performed on IIa subtypes, the biological and genetic characteristics of IId subtypes in China are not clear. We evaluated the infection and genetic characteristics of IId isolates in interferon-γ-knockout mice using qPCR to quantify oocyst shedding, histological examination to monitor pathological changes and comparative genomic analyses to identify infectivity and virulence-associated differences. Compared with the reference IIa isolate, mice infected with the IId isolates had significantly higher and longer oocyst shedding and lower body weight gain. In addition, the four IId isolates examined differed significantly in infectivity (as indicated by the median infective dose), oocyst shedding duration, and pathogenicity. Comparative genomic analysis indicated that the IId isolates had three more subtelomeric genes than the reference IIa isolate and 5385–5548 nucleotide substitutions, with the hypervariable genes mostly in two blocks on chromosome 1. In contrast, the four IId isolates differed from each other by 77–1,452 nucleotides, with virulence-associated sequence differences mainly in nine genes within a 28-kb block on chromosome 6. These data indicate the newly emerged *C*. *parvum* IId subtypes in China have high animal infectivity and unique genomic characteristics.

## Author summary

Cryptosporidiosis is the most important waterborne disease in industrialized nations and a primary cause of severe diarrhea in children in low- and middle-income countries. While the IIa subtype family of *Cryptosporidium parvum* is responsible for most zoonotic cryptosporidiosis, its IId subtype family has emerged in China in recent years. To understand the biological differences between the two major zoonotic subtype families, we have compared the infection patterns, virulence, and genetic characteristics of IIa and IId isolates using a newly established mouse model and whole genome sequencing. We have shown that IId isolates induce significantly higher infection intensity, longer infection duration, and more severe pathogenicity than the reference IIa isolate. They also have three more invasion-associated genes and substantial nucleotide sequence differences. In contrast, the four IId isolates with different virulence differ from each other mainly in sequences of nine genes within a small area on chromosome 6. We conclude that *C*. *parvum* isolates in China have high infectivity and unique genomic characteristics, and the productive infection model developed in the study should be useful in evaluations of potential therapeutics and studies of pathogenesis of *C*. *parvum*.

## Introduction

Cryptosporidiosis, caused by the protozoa *Cryptosporidium* spp., presents a significant public health problem in both industrialized nations and low- and middle-income countries [1]. Among the over 40 established *Cryptosporidium* species, *C*. *parvum* is the most important zoonotic species globally, with over 20 subtype families [2]. These subtype families differ in host range, with two of them, IIa and IId, being commonly found in both farm animals and humans. The former occurs widely in dairy calves in industrialized nations, while the latter has been found in lambs, goat kids, and dairy calves in some European and Mideast countries. Both are major causes of human cryptosporidiosis in these areas [3]. In China, IIa subtypes are largely absent, while IId subtypes are increasingly transmitted on dairy farms in the country. The latter are thought to be of rodent origin but have also been detected in other farm animals [4].

Thus far, virtually all biological characterizations and infection studies of *C*. *parvum* have been done with IIa subtypes, especially the widely used IOWA isolate [5]. Several murine models are commonly used in these studies, including neonatal [6], dexamethasone-suppressed [7], and immunodeficient mice, especially the interferon-γ knockout (GKO) mice [8, 9]. Virulence differs greatly among the IIa isolates examined, but many field isolates induce only transient infections with low oocyst shedding intensity and no apparent clinical signs [10, 11]. No infection studies have been conducted to assess possible differences in infectivity and patterns between the IIa and IId subtypes.

The biological characteristics of the newly emerged IId subtypes of *C*. *parvum* in China are not clear. Their broad host range [12], unique geographical distribution [13], and high virulence of some field IId isolates [14] indicate that IId subtypes could have genetic and biological characteristics significantly different from IIa subtypes. The objectives of the present study were to assess the infectivity of diverse IId isolates to GKO mice and compare the virulence of four field isolates using the commercial IIa-Waterborne isolate as a control. Comparative genomics analyses were conducted to identify infectivity- and virulence-associated genetic characteristics for improved understanding of the emergence of IId subtypes in China.

## Materials and methods

### Ethics statement

This study was performed in compliance with the Guide for the Care and Use of Laboratory Animals. The study protocol was approved by the Committee on the Ethics of Animal Use in Research, South China Agricultural University (No. 2019-C002, No. 2020-C007, No. 2020-C017 and No. 2020-C049).

### *C*. *parvum* isolates

The IIa-Waterborne isolate (designated as the IOWA isolate by the supplier but belonging to the IIaA17G2R1 subtype), which is widely used in vitro and in vivo studies of *C*. *parvum* [15, 16], was purchased from Waterborne, Inc. (New Orleans, LA, United States). The IIdA19G1-HN isolate originated from a dairy calf in Zhengzhou, Henan (HN), China and was a gift from Henan Agricultural University. The IIdA19G1-GD, IIdA20G1-HB and IIdA20G1-HLJ isolates were collected from dairy calves in Guangdong (GD), Hebei (HB) and Heilongjiang (HLJ) provinces, China, respectively (S1 Table). Among them, IIdA20G1-HB and IIdA20G1-HLJ were obtained from dairy farms with outbreaks of cryptosporidiosis that experienced significant mortality [14, 17]. Subtype identification was established using sequence analysis of the 60 kDa glycoprotein (*gp60*) gene. Oocysts of these isolates were passaged in calves or GKO mice, purified as described [18] and stored for less than three months prior to use in infection studies. Subtypes were confirmed before infections and every 5–7 days during infection periods. The number of oocysts used in inoculation was determined in triplicate by using a hemocytometer under a microscope for doses of ≥50 oocysts. For doses of ≤10 oocysts, oocysts were picked up individually under a microscope as described [19].

### Mice

Specific-pathogen-free GKO mice and wild-type (WT) mice with a C57BL/6 background were obtained from the Institute of Laboratory Animals Science, Chinse Academy of Medical Sciences. Mice were housed in individually ventilated cages under an isolation environment and supplied with sterilized diet, water, and bedding. Most infection studies were conducted with mice 6–8 weeks of age.

### Experimental infection of mice

All mice were determined to be *Cryptosporidium* free by fecal examination for three consecutive days prior to experimental infection as described [20]. All materials, such as cages, water bottles, and beddings, were autoclaved before reuse. The mice were used in five independent infection studies.

In the first infection study, the susceptibility of GKO and WT mice to the IId subtype was assessed. Three GKO mice and three WT littermates were inoculated by oral gavage with 10^5^ oocysts of the IIdA19G1-HN isolate. Oocyst shedding was examined by 18S-LC2 qPCR analysis of fecal samples collected daily [21]. The body weight of the animals was measured every other day.

In the second infection study, the infection patterns of isolates IId and IIa were compared. Eight GKO mice were randomly divided into three groups: the IIdA19G1-HN group (n = 3), IIa-Waterborne group (n = 3), and uninfected control group (n = 2, due to limited availability of GKO mice). Mice in the first two groups were inoculated by oral gavage with 10^6^ oocysts of IIdA19G1-HN or IIa-Waterborne. In addition, WT littermates (n = 3 for each group) were inoculated with IIdA19G1-HN or IIa-Waterborne oocysts for comparison. The oocyst shedding patterns and body weight were monitored for 40 days.

In the third infection study, the pathological changes induced by the IId subtype were examined. Nine GKO mice were randomly divided into three groups (n = 3 for each group): the IIdA19G1-HN group, IIa-Waterborne group and uninfected control group. Mice were sacrificed seven days after infection for the assessment of parasite burden and pathological changes in the ileum and colon using histology and scanning electron microscopy (SEM).

In the fourth infection study, the infection patterns and virulence of four IId isolates were compared. Twenty-four GKO mice were randomly divided into six groups (n = 4 for each group): IIdA19G1-HN, IIdA19G1-GD, IIdA20G1-HB, IIdA20G1-HLJ, IIa-Waterborne, and uninfected control groups. Animals in the five infection groups were inoculated with 2×10^5^ oocysts/mouse. The oocyst shedding pattern and body weight were monitored for 60 days.

In the fifth infection study, the median infective dose (ID_50_) of the four IId isolates was compared as described [22]. Forty-four 3- to 5-week-old GKO mice were randomly assigned to seven groups receiving different doses of oocysts: 1000 (n = 4), 100 (n = 4), 50 (n = 6), 10 (n = 8), 5 (n = 8), 1 (n = 8), and 0 (n = 6). Oocyst shedding in the inoculated mice was monitored for 30 days, and the ID_50_ of each isolate was calculated using logistic regression [23].

### Quantitation of oocyst shedding

DNA was extracted from 100 mg of feces using the FastDNA SPIN Kit for soil (MP Biomedicals, Santa Ana, CA, USA) [24]. The intensity of oocyst shedding in mice was assessed using SYBR Green-based 18S-LC2 qPCR [21]. The Cq values generated from the qPCR were used to calculate the number of oocysts per gram of feces (OPG) based on a standard curve generated using fecal samples spiked with known numbers of IIa-Waterborne oocysts. Fecal samples with log OPG values lower than 2.0 were considered negative for *C*. *parvum*. Nested PCR analysis of the SSU rRNA gene was used to confirm the negative results periodically.

### Histological examination and scanning electron microscopy (SEM)

The ileum and colon were collected from euthanized GKO mice and fixed in 4% paraformaldehyde for 24 hours, embedded in paraffin, cut into 4-μm-thick sections, and stained with hematoxylin and eosin using conventional procedures. The stained slides were examined under an Olympus BX53 (Olympus, Tokyo, Japan). Some tissues collected were prefixed in 2.5% glutaraldehyde, postfixed in 1% osmium tetroxide (OsO4), and examined by SEM using an EVO MA 15/LS 15 (Carl Zeiss Microscopy GmbH, Jena, Germany).

### Comparative genomic analysis of *C*. *parvum* isolates

Genomic DNA was extracted from the purified oocysts and sequenced using Illumina NextSeq 500 analysis of 150-bp paired-end libraries (Illumina, San Diego, CA, USA) as described [25]. The genomes were assembled for the analysis of gene insertions and deletions (indels) and single nucleotide variants (SNVs) among IId and IIa isolates as described [26]. Highly polymorphic genes between IIa and IId isolates or among IId isolates were identified using the mean + 3 standard deviation values of SNVs. Maximum likelihood (ML) trees were constructed based on genome-wide SNVs to assess the phylogenetic relationships among isolates using RAxML-NG v1.0.0 [27], the GTR+G model, and 1000 bootstrap replicates.

### Statistical analysis

Student’s *t*-test was used to compare the oocyst shedding intensity among groups. Differences were considered significant at *P* ≤ 0.05.

## Results

### Susceptibility of GKO mice to the *C*. *parvum* IId subtype family

In the initial evaluation of the susceptibility of GKO mice to the IId subtype family and the establishment of an infection model, all mice inoculated with 10^5^ oocysts of IIdA19G1-HN became infected. The subtype identifications confirmed that the oocysts inoculated and produced after infection belonged to IIdA19G1. Oocyst shedding increased gradually, reached a peak (OPG over 10^6^) on Day 12, and was maintained at a high shedding level until the study was terminated on Day 30 (S1B Fig). In contrast, WT mice displayed transient infection, with oocyst shedding reaching the peak (OPG approximately 10^4^) on Day 5. The oocyst shedding intensity in GKO mice had been significantly higher than that in WT mice since Day 11 (*t* = 11.764, *P* = 0.007). No oocysts were detected in WT mice by qPCR and nested PCR after Day 15.

### Performance of the GKO model of the *C*. *parvum* IId subtype family

**Fig 1:**
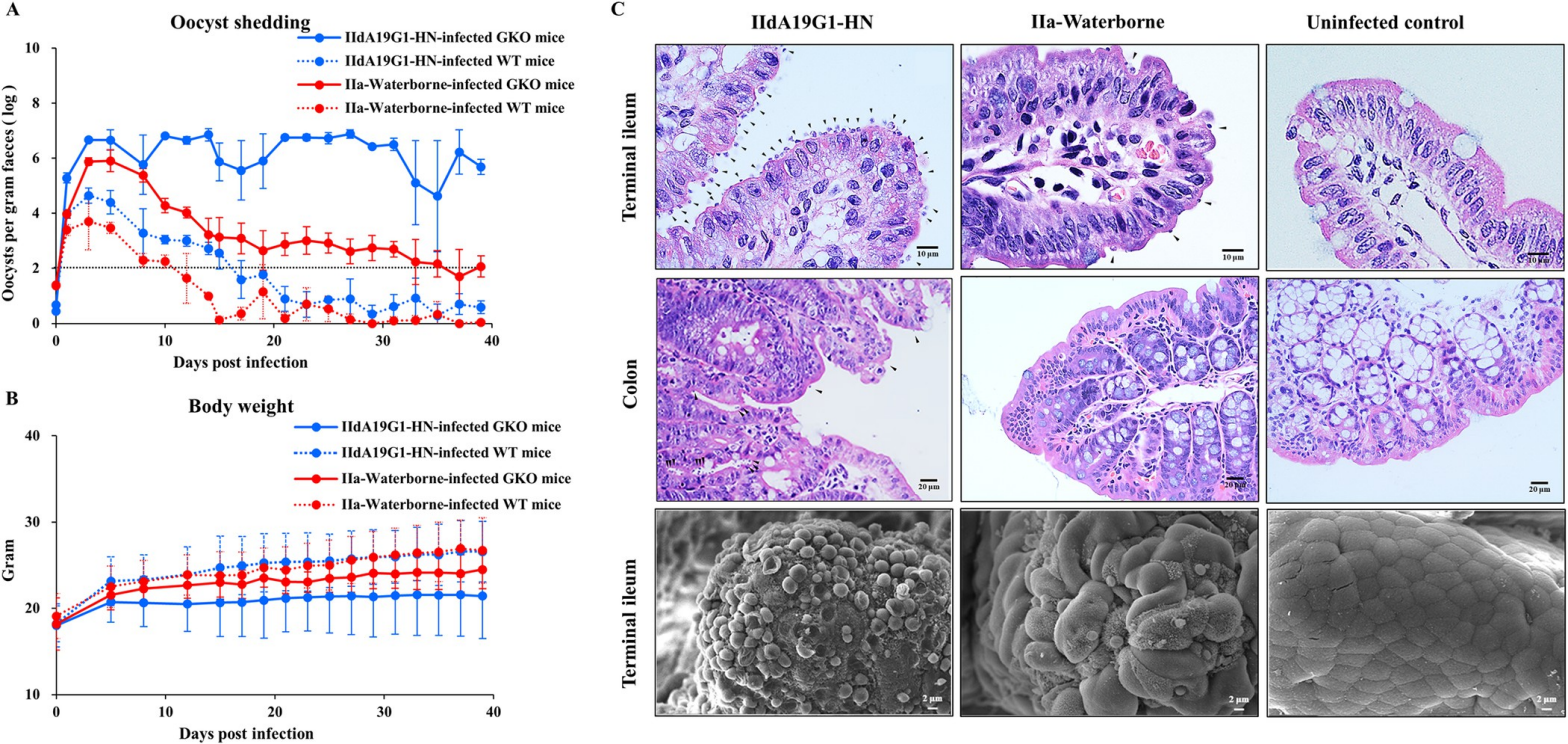
The interferon-γ-knockout (GKO) mouse model is susceptible to *Cryptosporidium parvum* isolates and can distinguish isolate-specific differences at the pathological level. (A) Oocyst shedding patterns of IIdA19G1-HN-infected GKO mice, IIdA19G1-HN-infected wild-type (WT) mice, IIa-Waterborne-infected GKO mice, and IIa-Waterborne-infected wild-type (WT) mice. Fecal samples were considered oocyst-negative when the number of OPG (log) was lower than 2.0 (indicated with a dotted line). (B) Body weight changes in these mice. (C) Hematoxylin and eosin microscopy (upper and middle panels) and scanning electron microscopy (lower panel) images of the terminal ileum and colon of GKO mice infected with IIdA19G1-HN and IIa-Waterborne isolates in comparison with the uninfected control (parasites are indicated with arrowheads).

The infection pattern and virulence were further compared between IIdA19G1-HN and IIa-Waterborne subtypes. The subtype identifications indicated that the subtypes of oocysts produced in GKO mice were consistent with those of inoculated oocysts. In GKO mice, IIdA19G1-HN induced longer oocyst shedding duration and significant higher oocyst shedding intensity (Day 10, *t* = 11.397, *P* = 0.008) than IIa-Waterborne, with OPG at approximately 10^6^ throughout the study. In contrast, oocyst shedding in the IIa-Waterborne-infected GKO mice started to decline after reaching a peak on Day 8 and became negative before Day 40 (Fig 1A). GKO mice infected with IIdA19G1-HN had slightly reduced body weight gains compared with those infected with IIa-Waterborne during the entire period (*P* = 0.241–0.474) (Fig 1B). They further had ruffled hair and inactivity. Mice infected with IIa-Waterborne appeared normal.

In WT mice, both IIdA19G1-HN and IIa-Waterborne induced transient infections. The oocyst shedding intensity in GKO mice infected with IIdA19G1-HN had been significantly higher than that in WT mice since Day 10 (*t* = 62.544, *P* = 0.000). Likewise, in infections with IIa-Waterborne, GKO mice had significantly higher oocyst shedding intensity than WT mice since Day 5 (*t* = 12.200, *P* = 0.007). Oocyst shedding peaked on Day 4 for both subtypes in WT mice, but IIdA19G1-HN induced slightly longer infection and higher oocyst shedding than IIa-Waterborne (*P* = 0.084–0.502). All mice appeared normal and had similar body weight gains during the entire period (Fig 1).

### Parasite burdens in intestinal tissues of GKO mice infected with *C*. *parvum*

In the third infection study, the parasite burden in intestinal tissues was compared between IIa- and IId-infected GKO mice. At Day 7, the parasite burden was higher in the ileum of mice infected with IIdA19G1-HN than in those infected with IIa-Waterborne (statistical analysis was not done). Although edema was observed, the architecture of the intestinal villi remained intact. A small number of parasites were detected in the colon of IIdA19G1-HN-infected mice (Fig 1C upper and middle panels). SEM of the ileal tissue showed abundant parasites on the intestinal epithelium of IIdA19G1-HN-infected mice but many fewer parasites in the ileum of IIa-Waterborne-infected mice (Fig 1C lower panel). Uninfected mice showed no parasites in the intestinal tissues.

### Differences in infection patterns and virulence among IId isolates

**Fig 2:**
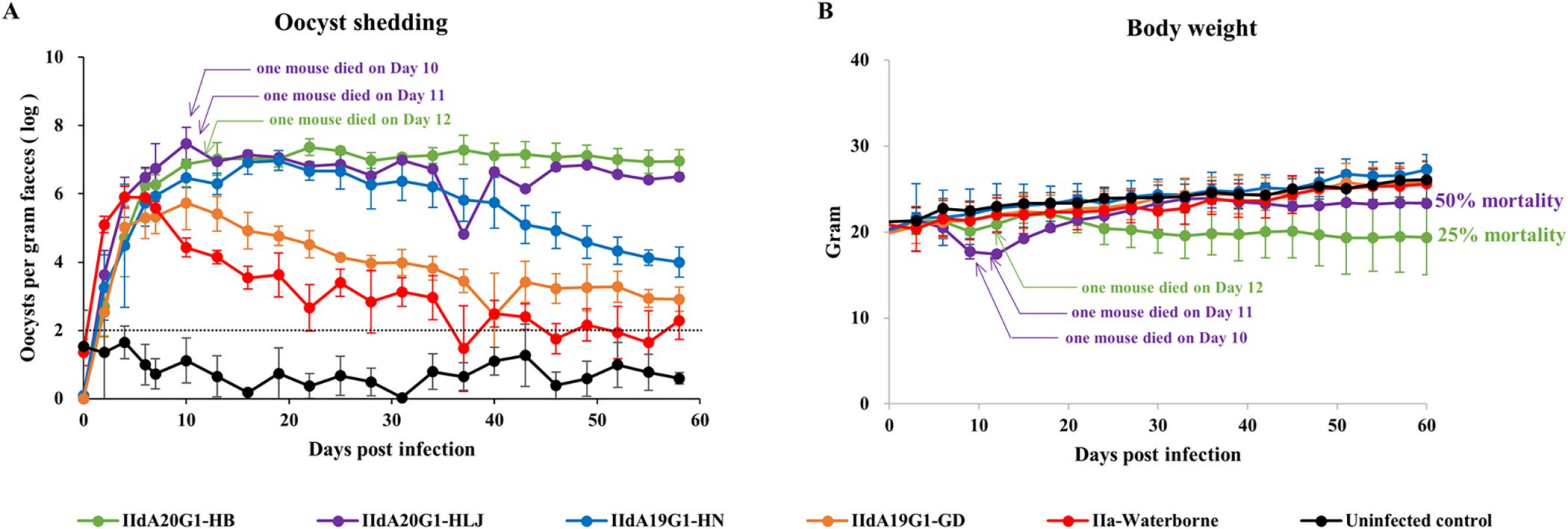
Virulence differed among *Cryptosporidium parvum* IId isolates. (A) Oocyst shedding patterns of interferon-γ-knockout (GKO) mice infected with four IId isolates and the reference IIa-Waterborne isolate. The detection threshold is indicated with a dotted line. (B) Body weight changes in GKO mice infected with four IId isolates and the IIa-Waterborne isolate.

The infection patterns and virulence of IId field isolates were compared in the fourth infection study in GKO mice. The subtype identifications confirmed that the subtypes of oocysts produced in GKO mice were consistent with those of inoculated oocysts. Among the four isolates examined, IIdA20G1-HB and IIdA20G1-HLJ were similar in oocyst shedding duration and intensity (Fig 2A). IIdA20G1-HB induced significantly higher oocyst shedding intensity than IIdA19G1-HN after Day 40 (Day 43, *t* = 4.651, *P* = 0.006) and IIdA19G1-GD after Day 10 (Day 13, *t* = 3.615, *P* = 0.016). IIdA20G1-HB and IIdA20G1-HLJ also induced longer oocyst shedding duration than IIdA19G1-HN and IIdA19G1-GD. Among them, IIdA20G1-HB and IIdA20G1-HLJ produced over 10^7^ OPGs in GKO mice for more than 8 weeks until the termination of the infection study. In contrast, the OPG values in GKO mice infected with IIdA19G1-HN persisted at approximately 10^6^ for only 4 weeks. The OPG values in GKO mice infected with IIdA19G1-GD started to decline after reaching a peak of 10^6^ on Day 10. Overall, the oocyst shedding intensity and duration induced by IIdA19G1-GD were just slightly above those induced by IIa-Waterborne (Fig 2A).

GKO mice infected with IIdA20G1-HB had no body weight gains during the study period. Body weight in IIdA20G1-HLJ-infected mice decreased during early infection but recovered somewhat subsequently. In contrast, the IIdA19G1-HN- and IIdA19G1-GD-infected groups had body weight gains similar to those of uninfected controls (Fig 2B). Mice infected with IIdA20G1-HB and IIdA20G1-HLJ started to have sticky fecal pellets, rough hair, arched back, and inactivity after Day 6, while those infected with IIdA19G1-HN, IIdA19G1-GD, and IIa-Waterborne appeared largely normal. One and two of the four mice in the IIdA20G1-HB- and IIdA20G1-HLJ-infected groups died of cryptosporidiosis, respectively.

### Differences in infectivity among four IId isolates

**Fig 3:**
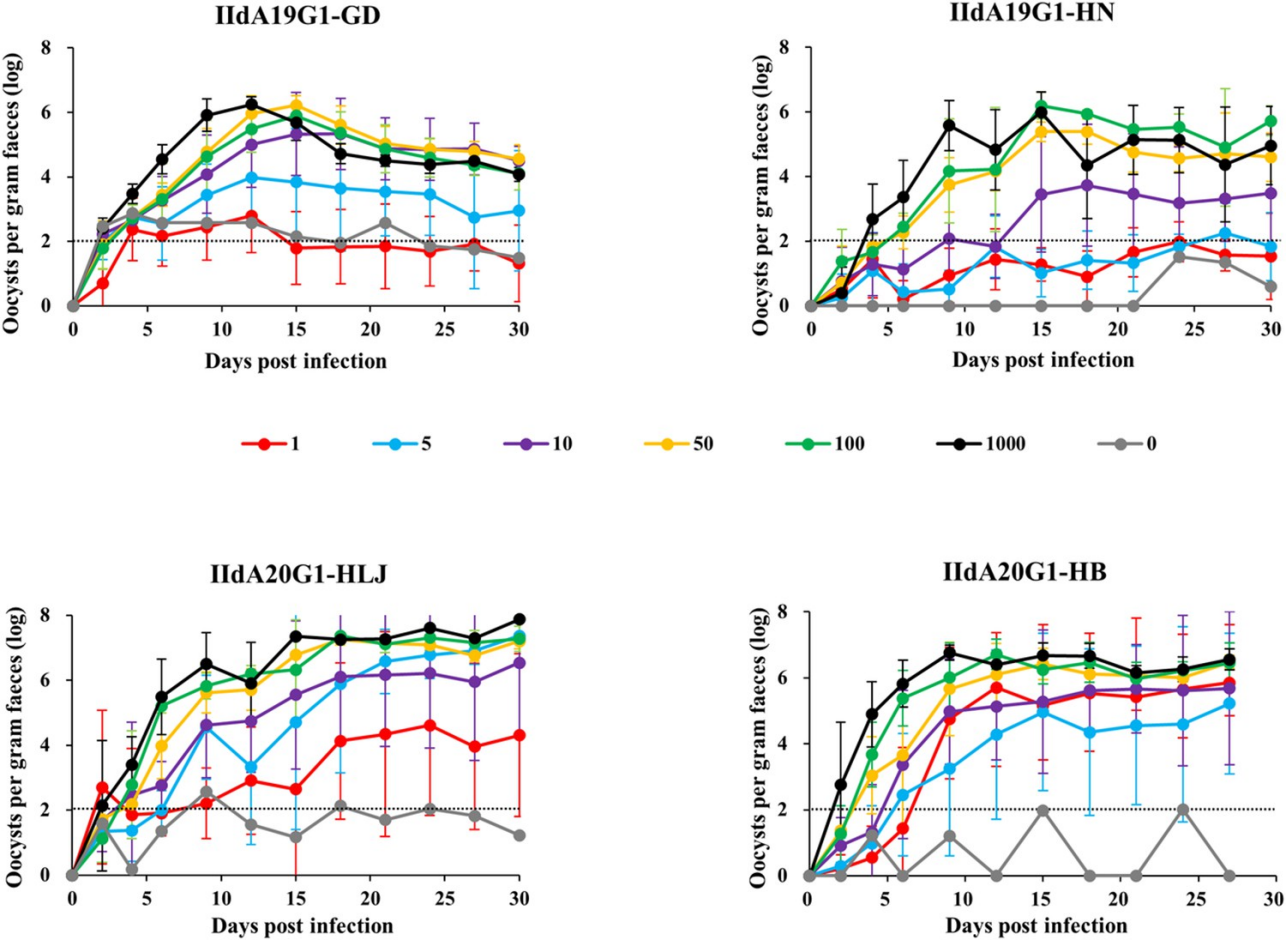
Oocyst shedding patterns of interferon-γ-knockout (GKO) mice infected with different doses of four *Cryptosporidium parvum* IId isolates. The detection threshold is indicated with a dotted line.

In the fifth infection study, the ID_50_ of four IId isolates was measured. The subtype identifications confirmed that the subtypes of oocysts produced in GKO mice were consistent with those of inoculated oocysts. Among them, inoculations with single IIdA20G1-HB and IIdA20G1-HLJ oocysts induced infection in most GKO mice, with the calculated ID_50_ values being lower than 1 oocyst. In contrast, more oocysts were needed to induce infections with IIdA19G1-GD and IIdA19G1-HN, with ID_50_ values of approximately five and seven oocysts, respectively (Table 1). For each isolate, the prepatent period and the time to peak oocyst shedding were longer when lower oocyst doses were used in inoculation (Fig 3). For IIdA20G1-HLJ, 3/4, 2/4, 2/6, 2/8, 3/8, and 2/8 mice inoculated with 1000, 100, 50, 10, 5 and 1 oocyst died on days 11–17 post infection, respectively. None of the mice infected with other IId isolates died during the ID_50_ study. At the peak of oocyst shedding, IIdA20G1-HB and IIdA20G1-HLJ induced higher OPGs than IIdA19G1-GD and IIdA19G1-HN. For instance, in the 50-oocyst groups which resulted in 100% infection rate for the four isolates, the peak OPG (log) of IIdA20G1-HB, IIdA20G1-HLJ, IIdA19G1-GD and IIdA19G1-HN were 6.49 ± 0.20, 7.26 ± 0.19, 6.22 ± 0.29 and 5.39 ± 0.40, respectively. Therefore, IIdA20G1-HLJ induced significantly higher peak OPG (log) than IIdA19G1-HN (*t* = 8.711, *P* = 0.000) and IIdA19G1-GD (*t* = 6.243, *P* = 0.000). IIdA20G1-HB also induced significantly higher peak OPG (log) than IIdA19G1-HN (*t* = 5.874, *P* = 0.002) but only slightly higher OPG (log) than IIdA19G1-GD (*t* = 1.719, *P* = 0.146). OPG values in mice infected with IIdA19G1-GD started to decline after the peak around Day 15 (Fig 3).

**Table 1:** Infectivity of four IId isolates of *Cryptosporidium parvum* in interferon-γ-knockout mice.

Inoculation dose	No. of mice becoming positive/No. of mice inoculated (infection rate in %)			
	IIdA19G1-GD	IIdA19G1-HN	IIdA20G1-HLJ	IIdA20G1-HB
1000	4/4 (100)	4/4 (100)	4/4 (100)	4/4 (100)
100	4/4 (100)	4/4 (100)	4/4 (100)	4/4 (100)
50	6/6 (100)	6/6 (100)	6/6 (100)	6/6 (100)
10	7/8 (88)	6/8 (75)	7/8 (88)	7/8 (88)
5	4/8 (50)	1/8 (13)	8/8 (100)	5/8 (63)
1	0/8 (0)	1/8 (13)	5/8 (63)	7/8 (88)
0	0/6 (0)	0/6 (0)	0/6 (0)	0/6 (0)
ID_50_^a^	5.1 oocysts	7.1 oocysts	0.6 oocysts	0.1 oocysts

^a^ ID_50_, the median infective dose, is the dose that is expected to cause infection in 50% mice as calculated from the data using logistic regression.

### Genomic differences between IIa and IId subtype families

**Fig 4:**
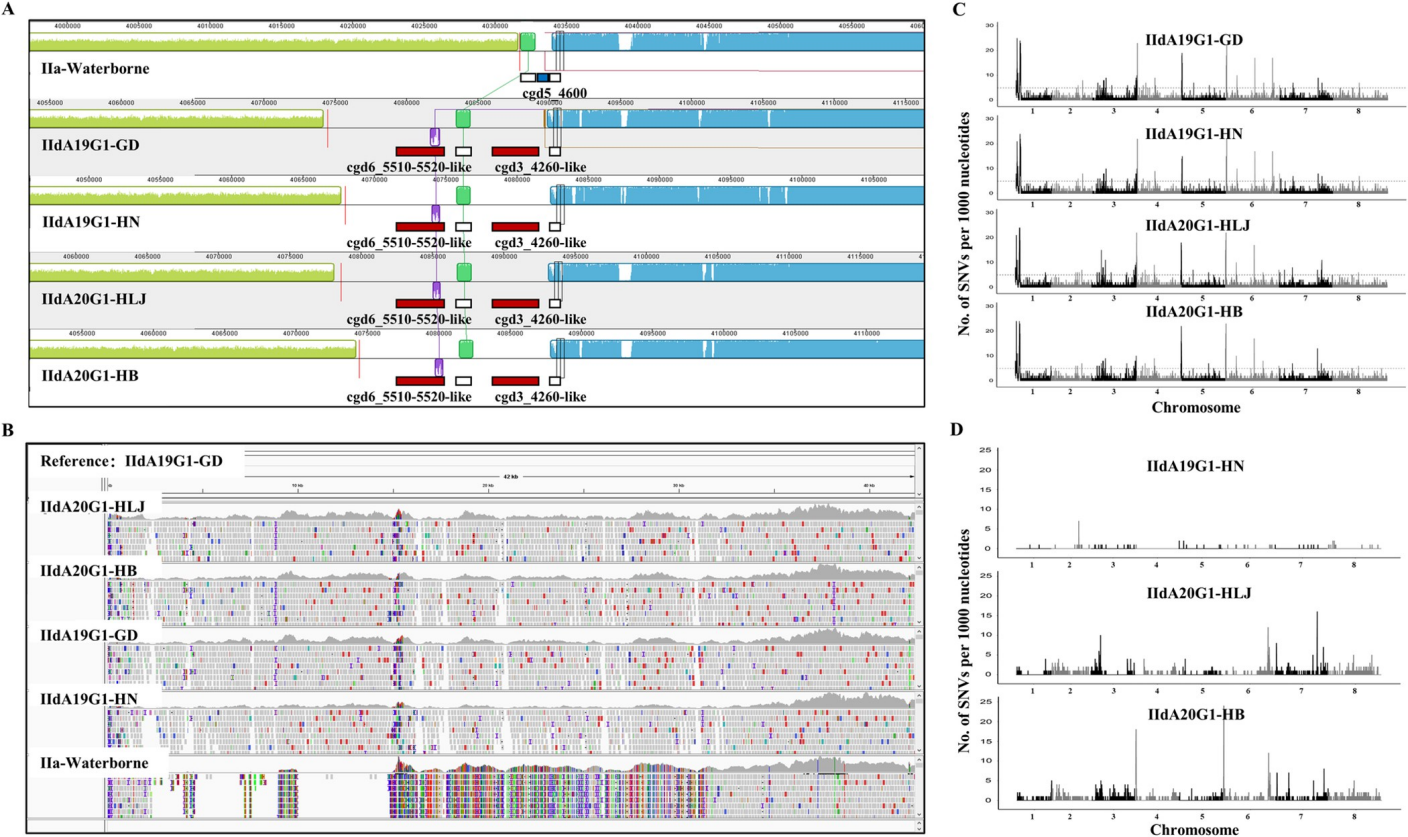
Genetic differences among *Cryptosporidium parvum* isolates. (A) Insertions and deletion in four IId genomes at the 5′ end of chromosome 5 compared with the genome of IIa-Waterborne (indicated by small red and blue blocks, respectively). (B) Confirmation of the gene insertions in four IId genomes at the 5′ end of chromosome 5 using read mapping. (C) Distribution of single nucleotide variants in IIdA19G1-GD, IIdA19G1-HN, IIdA20G1-HLJ and IIdA20G1-HB compared with the IIa-Waterborne genome. (D) Distribution of single nucleotide variants in IIdA19G1-HN, IIdA20G1-HLJ and IIdA20G1-HB compared with the IIdA19G1-GD genome.

Approximately 7.3–29.4 million 150-bp paired-end reads were obtained from each of the five *C*. *parvum* isolates, generating genome assemblies of 9.05–9.12 Mb in 106–203 contigs (S1 Table). The IId and IIa isolates differed in the copy numbers of subtelomeric genes encoding three protein families. Compared with IIa-Waterborne, IId isolates have one additional cgd3_10-like SKSR gene at the 3′ end of chromosome 3, one cgd3_4260-like insulinase gene and one cgd6_5510-5520-like insulinase gene at the 5′ end of chromosome 5, and the deletion of the cgd5_4600 gene encoding a MEDLE secretory protein (Table 2 and Fig 4A). The indels on chromosome 5 in IId isolates were confirmed by read mapping (Fig 4B) and PCR analysis (S2 Table and S2 Fig).

**Table 2:** Copy number variations in subtelomeric genes between IIa and IId isolates of *Cryptosporidium parvum*.

Chromosome	*Cryptosporidium parvum* isolates					
	IIdA19G1-GD	IIdA19G1-HN	IIdA20G1-HLJ	IIdA20G1-HB	IIa-Waterborne	Annotation
3	cgd3_10 paralog	cgd3_10 paralog	cgd3_10 paralog	cgd3_10 paralog	-	SKSR protein
5	cgd6_5510–5520	cgd6_5510–5520	cgd6_5510–5520	cgd6_5510–5520	-	insulinase-like protease
5	cgd3_4260 paralog	cgd3_4260 paralog	cgd3_4260 paralog	cgd3_4260 paralog	-	insulinase-like protease
7	-	-	-	-	cgd5_4600	MEDLE family of secreted protein

The comparative genomic analysis also identified 5385–5548 SNVs between the IIa-Waterborne and IId isolates, mostly on chromosomes 1, 3, 6 and 7 (Fig 4C). Forty-seven highly polymorphic protein-coding genes were identified between the IIa-Waterborne isolate and the four IId isolates (S3 Fig), and 31 of them were common in four IId isolates, mainly in several areas on chromosomes 1 and 6 (S3 Table). Notably, two blocks of 20 kb and 26 kb on chromosome 1 containing 6 and 12 genes, respectively, were divergent between the IIa-Waterborne isolate and the four IId isolates.

### Genomic differences among IId isolates

Compared with the genome of IIdA19G1-GD, there were 77, 1158, and 1452 SNVs in the genomes of IIdA19G1-HN, IIdA20G1-HLJ and IIdA20G1-HB, respectively (S4 Table). The IIdA20G1-HB-specific SNVs were mainly in the 5′ subtelomeric areas of chromosomes 4 and 6, and many were from noncoding regions (Fig 4D and S4 Table). In phylogenetic analysis of genome-wide SNVs, IIdA19G1-GD and IIdA19G1-HN clustered together, while IIdA20G1-HB and IIdA20G1-HLJ formed divergent branches (S4 Fig). Forty-six protein-coding genes were highly polymorphic among IId isolates (S5 Table), including 13 shared by IIdA20G1-HLJ and IIdA20G1-HB. Nine of the 13 genes with high virulence-specific sequences were in a 28-Kb block on chromosome 6 (Table 3).

**Table 3:** Potential virulence-associated genes among four IId Isolates of *Cryptosporidium parvum*.

Chromo-some	Gene ID in *C*. *parvum* IOWA	Annotation	Gene length (bp)	No. of single nucleotide variants (SNVs)^a^		
				IIdA19G1-HN	IIdA20G1-HLJ	IIdA20G1-HB
2	cgd2_750	WD40 repeat containing protein	1269	-	4	4
3	cgd3_360	Uncharacterized protein	393	-	2	2
5	cgd5_4610	MEDLE gene family	716	-	4	3
6	cgd6_4740	Uncharacterized protein	834	-	3	3
6	cgd6_4750	Splicing factor 3B subunit 1	3096	-	19	19
6	cgd6_4770	Torus/RNA recognition motif domain containing protein	1029	-	3	3
6	cgd6_4780	Uncharacterized protein	1221	-	4	5
6	cgd6_4790	Uncharacterized protein with B-box-type zinc finger	3477	-	9	8
6	cgd6_4800	P-loop containing nucleoside triphosphate hydrolase	1320	-	5	5
6	cgd6_4830	DEAD/DEAH box helicase	1632	-	4	4
6	cgd6_4840	Serine protease, subtilase family	4051	-	7	7
6	cgd6_4850	Pre-mRNA-splicing factor 19 with U box domain	1656	-	5	5
8	cgd8_2400	Uncharacterized protein	750	-	2	2

^a^ Compared with the reference genome of IIdA19G1-GD.

## Discussion

We conducted a comparative assessment of the infectivity, infection patterns, and genetic characteristics of several *C*. *parvum* IId isolates in GKO mice. The data generated showed high infectivity of the four field isolates examined, as indicated by the low infective doses. In addition, they induced significantly higher intensity and long duration of oocyst shedding in these animals than the reference IIa isolate. Differences in infectivity and virulence have also been observed among the IId isolates. The results of comparative genomic analyses have provided clues on genetic determinants for biological differences between IIa and IId isolates and virulence differences among IId isolates, which could shed light on the potential contribution of biological and genetic factors to the recent emergence of *C*. *parvum* IId subtypes in China.

The IId subtype family appears to differ biologically from the IIa subtype family of *C*. *parvum*. In industrialized nations, IIa subtypes are prevalent in dairy cattle, while IId subtypes are mainly found in small ruminants in some European and Middle East countries [3]. However, IId subtypes are the dominant subtypes in dairy cattle in China, where they have been found in a broad range of other hosts [4]. The difference in host ranges between IIa subtypes in industrialized nations and IId subtypes in China could be a reflection of their differences in infectivity and virulence in GKO mice in the present study. The four IId isolates examined showed significantly higher and longer oocyst shedding than the reference IIa-Waterborne isolate. They in turn have led to lower weight gain, malnutrition (indicated by rough hair coat), and some mortality in IId-infected mice. In comparison, IIa-Waterborne induced only transient and light oocyst shedding with no apparent clinical signs. Previously, it was suggested that in dairy calves in Sweden, IId subtypes might be less virulent than IIa subtypes [28]. This does not appear to be the case with Chinese IId isolates in GKO mice. IId subtypes have been responsible for outbreaks of severe diarrhea in dairy calves in China with high mortality [14, 17, 29, 30].

The four IId isolates examined in this study also differ in infectivity and virulence. In this study, IIdA20G1-HLJ and IIdA20G1-HB induced significantly higher intensity and longer oocyst shedding than IIdA19G1-HN and IIdA19G1-GD. This correlated with the lower ID_50_ of these two isolates. Because of the higher parasite burden, mice infected with IIdA20G1-HLJ and IIdA20G1-HB had reduced body weight, arched back, inactivity, and some mortality. In contrast, the intensity and duration of oocyst shedding in mice infected with IIdA19G1-GD were just slightly higher than those in mice infected with IIa-Waterborne and were not associated with any clinical signs. Differences in infectivity and virulence are well known among IIa isolates [10, 31, 32]. In the present study, the two IId isolates with lower infective doses, higher and longer oocyst shedding, and higher virulence in GKO mice were from dairy farms with cryptosporidiosis outbreaks and the associated mortality, indicating that there is correlation in the virulence of *C*. *parvum* between calves and immunocompromised mice.

Copy number variations in subtelmeric genes could be responsible for differences in infectivity between IId and IIa isolates. Compared with the reference IIa isolate, IId isolates had one additional SKSR gene, two additional protease genes, and one less MEDLE gene. Duplication or deletion of these subtelomeric genes was suggested to be involved in differences in host specificity among genetically related *Cryptosporidium* spp. [33]. These differences might contribute to differences in host preference and infection patterns between the IIa and IId subtypes. Sequence differences in other functional proteins could also be involved in the biological differences between the IIa and IId subtypes. The >5000 SNVs between the IIa-Waterborne isolate and the four IId isolates were mostly located on chromosomes 1 and 6. They include genes encoding mucin glycoproteins, SKSR proteins, and other secretory proteins, which have been identified as secreted pathogenesis determinants of *Cryptosporidium* [34].

Sequence differences within a small region of chromosome 6 appear to be associated with the virulence and infectivity of IId isolates. The results of comparative genomic analysis indicate that isolate-specific SNVs instead of large indels are the main genomic differences among IId isolates. In particular, the virulent IIdA20G1-HLJ and IIdA20G1-HB isolates differed from the low-virulence isolates IIdA19G1-GD and IIdA19G1-HN mostly in the nucleotide sequences of 13 genes. As nine of the 13 genes involved are in a 28-kb region within chromosome 6, genetic determinants for virulence in IId isolates could be located in this area. It is possible that one gene in this region is involved in virulence, while other sequences could be just piggybacked as a result of a selective sweep. Additional genetic studies with the CRISPR/Cas9 technique are necessary to identify the exact virulence determinant in *C*. *parvum* IId isolates.

The IId-GKO infection model should be useful in the long-term maintenance and production of oocysts and the development of new therapeutics against cryptosporidiosis. Compared to the transient infection and low oocyst shedding intensity of IIa isolates examined thus far by various researchers, the IId isolates in the study have induced persistent infection with high oocyst shedding intensity in GKO mice. This would allow the stable provision of large numbers of oocysts for biological studies of the pathogen. The persistent infection would facilitate the evaluation of efficacy of candidate compounds, as the self-cure of the infection or death of infected GKO mice within a few days of experimental infection with IIa subtypes makes the reliable assessment of their anti-cryptosporidial effects difficult.

In summary, we have demonstrated that significant biological differences are present between the IIa and IId subtypes of *C*. *parvum*. They are reflected by differences in whole genome sequences and infectivity and virulence in infection of laboratory mice. While copy number variations in subtelomeric genes encoding several secretory proteins are associated with the biological differences between the IIa and IId subtypes, subtle sequence differences in a 28-kb region of chromosome 6 could be responsible for virulence differences among IId isolates. These data provide rich targets for advanced studies of genetic determinants of infectivity and virulence of the pathogens. The IId-GKO infection model developed in the study should be also useful in evaluations of potential therapeutics and studies of pathogenesis of *C*. *parvum*.

## Supporting information

S1 Fig Initial experimental infection of *Cryptosporidium parvum* IIdA19G1-HN isolate in Interferon-γ-knockout (GKO) mice. (A) Differential interference contrast microscopy of IIdA19G1-HN oocysts, bar = 10 μm. (B) Oocyst shedding pattern of GKO mice and wild-type (WT) mice infected with IIdA19G1-HN isolate.
(TIF)

S2 Fig Confirmation of major insertion at the 5’ end of chromosome 5 in *Cryptosporidium parvum* IId genomes by PCR. Among the IId and IIa specimens analyzed, all the four IId specimens produced the expected 748 bp PCR product. M, size marker in 2000 bp; lane 1, IIdA19G1-HN; lane 2, IIdA19G1-GD; lane 3, IIdA20G1-HB; lane 4, IIdA20G1-HLJ; lane 5, IIa-Waterborne; lane 6, negative control for primary PCR; and lane 7, negative control for secondary PCR.
(TIF)

S3 Fig Venn diagram of highly polymorphic protein-coding genes shared by IIdA19G1-GD, IIdA19G1-HN, IIdA20G1-HLJ and IIdA20G1-HB with comparison to assembled IIa-Waterborne genome. (TIF)

S4 Fig **Phylogenetic relationship of the *Cryptosporidium parvum* IId and IIa isolates** based on maximum likelihood analysis of (A) nucleotide sequences of 60 kDa glycoprotein (*gp60*) gene and (B) genome-wide single nucleotide variants (SNVs) with comparison to the assembled IIdA19G1-GD genome. The numbers on the branches are percent bootstrapping values from 1000 replicates.
(TIF)

S1 Table Information of five *Cryptosporidium parvum* isolates examined in this study and summary of whole genome sequencing (WGS) data. (DOCX)

S2 Table Primers of nested PCR for confirmation of the major insertion at the 5’ end of chromosome 5 in *Cryptosporidium parvum* IId genomes. (DOCX)

S3 Table Common highly polymorphic genes in *Cryptosporidium parvum* IId genomes with comparison to the genome of IIa-Waterborne. (DOCX)

S4 Table Summary of single nucleotide variants (SNVs) in IId genomes sequenced in this study compared with the genome of IIdA19G1-GD. (DOCX)

S5 Table Highly polymorphic genes between *Cryptosporidium parvum* IIdA19G1-GD and the other three IId isolates, IIdA19G1-HN, IIdA20G1-HLJ and IIdA20G1-HB. (DOCX)
