# Supplementary figures and images for: High infectivity and unique genomic sequence characteristics of Cryptosporidium parvum in China
Source: PLoS Negl Trop Dis. 2022 Aug 22;16(8):e0010714. doi: 10.1371/journal.pntd.0010714 (PMC9436107; doi:10.1371/journal.pntd.0010714)

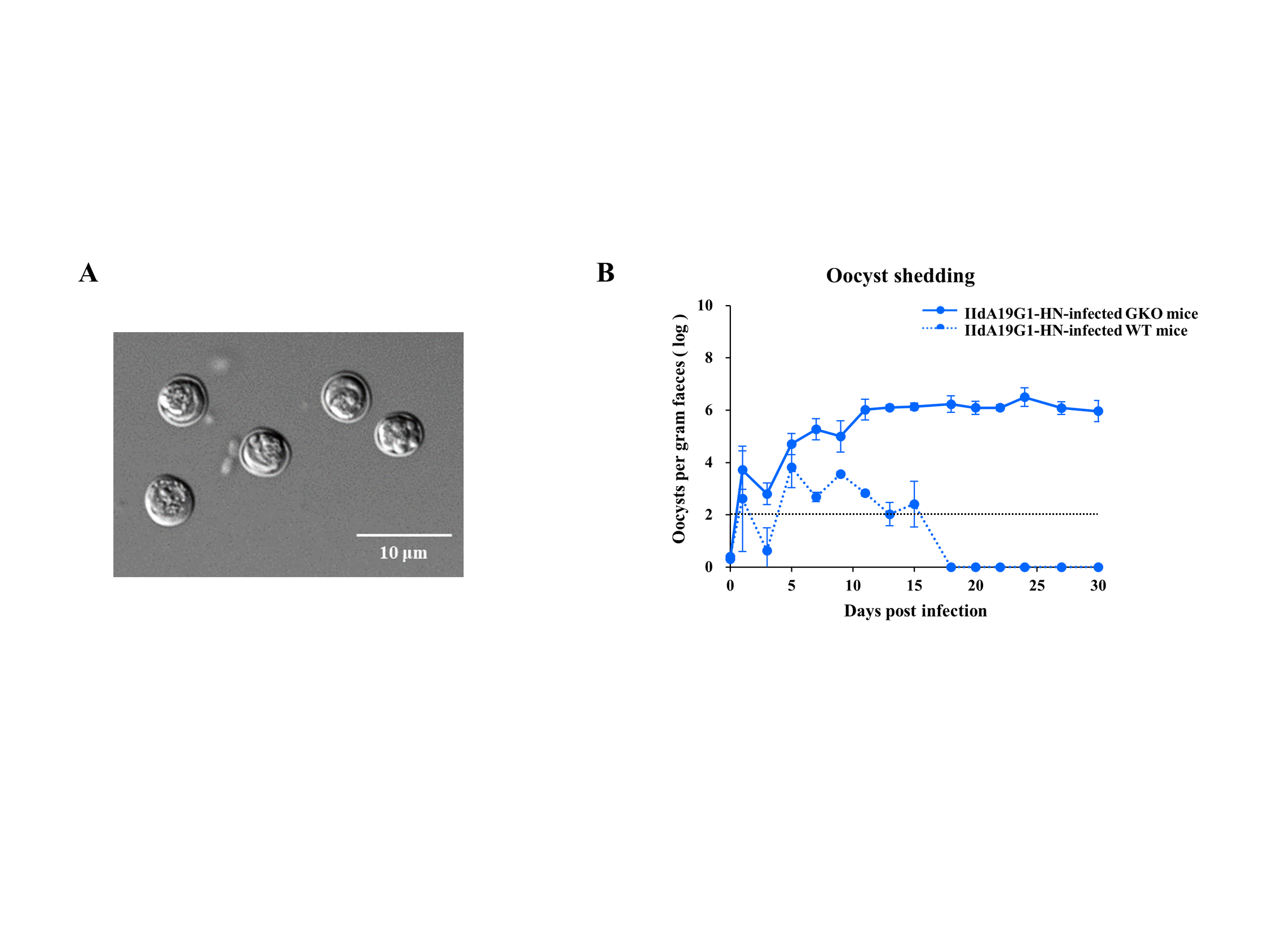

Supplement: S1 Fig — (A) Differential interference contrast microscopy of IIdA19G1-HN oocysts, bar = 10 μm. (B) Oocyst shedding pattern of GKO mice and wild-type (WT) mice infected with IIdA19G1-HN isolate. (TIF) [file pntd.0010714.s001.tif]

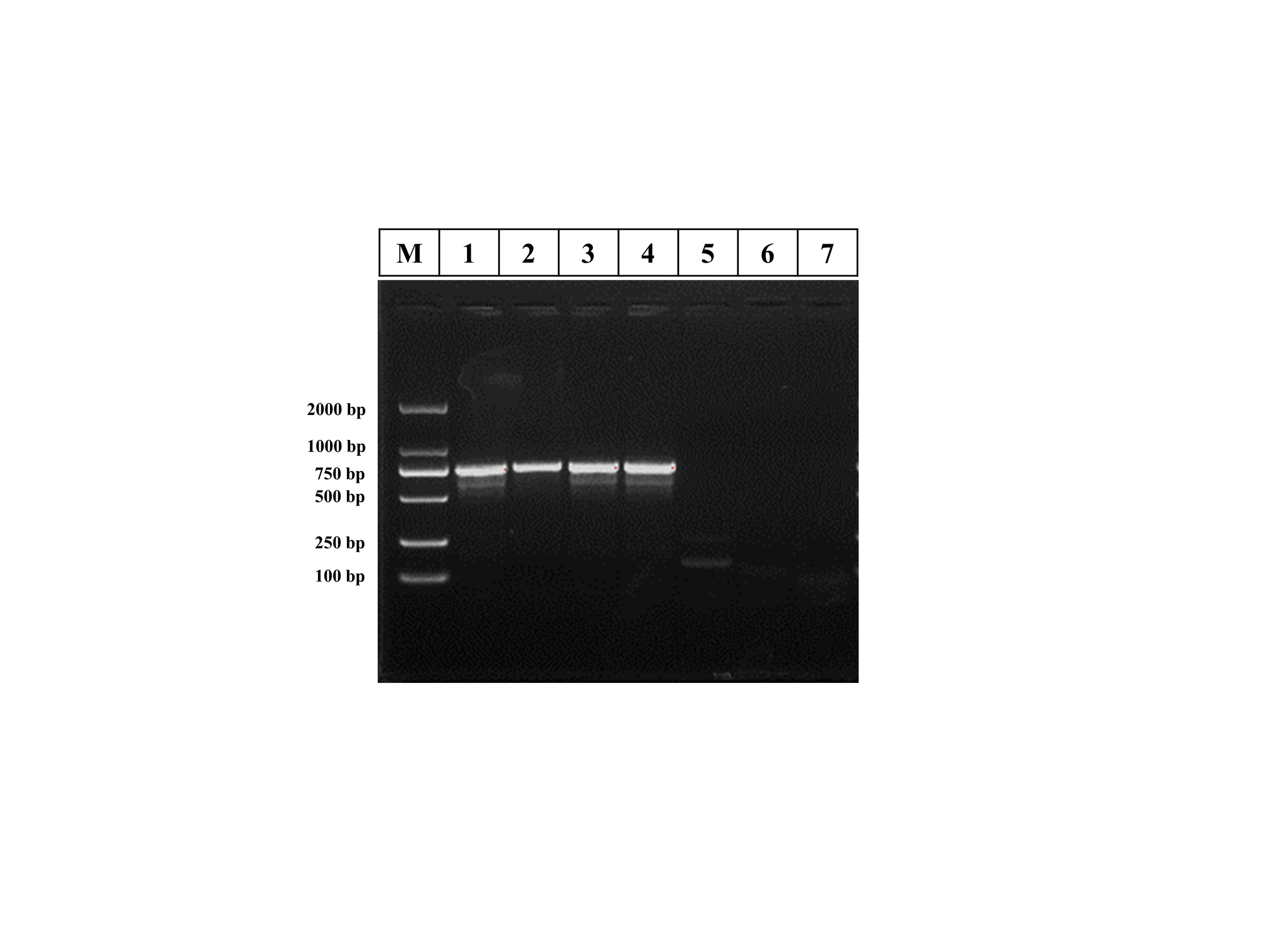

Supplement: S2 Fig — Among the IId and IIa specimens analyzed, all the four IId specimens produced the expected 748 bp PCR product. M, size marker in 2000 bp; lane 1, IIdA19G1-HN; lane 2, IIdA19G1-GD; lane 3, IIdA20G1-HB; lane 4, IIdA20G1-HLJ; lane 5, IIa-Waterborne; lane 6, negative control for primary PCR; and lane 7, negative control for secondary PCR. (TIF) [file pntd.0010714.s002.tif]

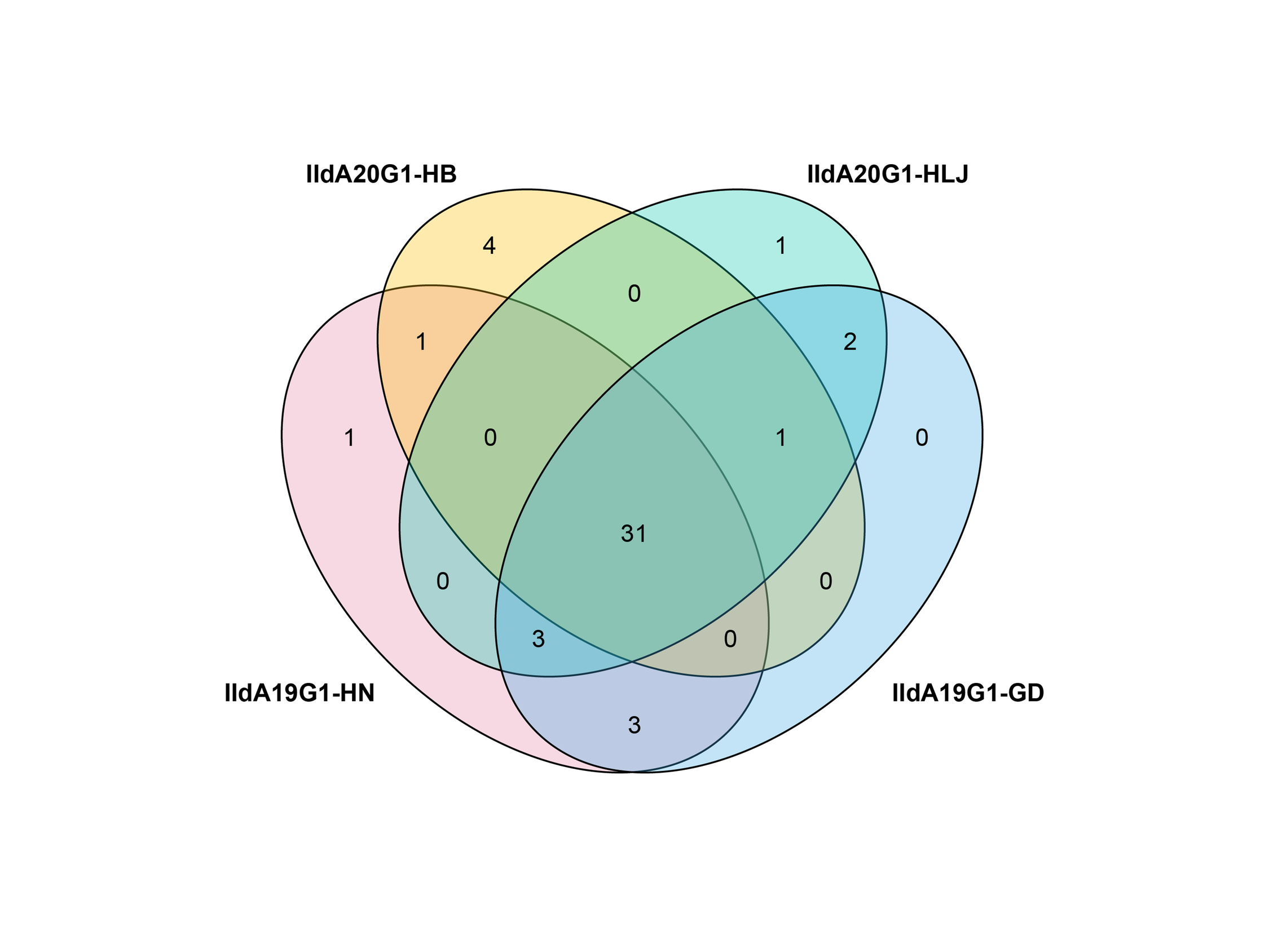

Supplement: S3 Fig — (TIF) [file pntd.0010714.s003.tif]

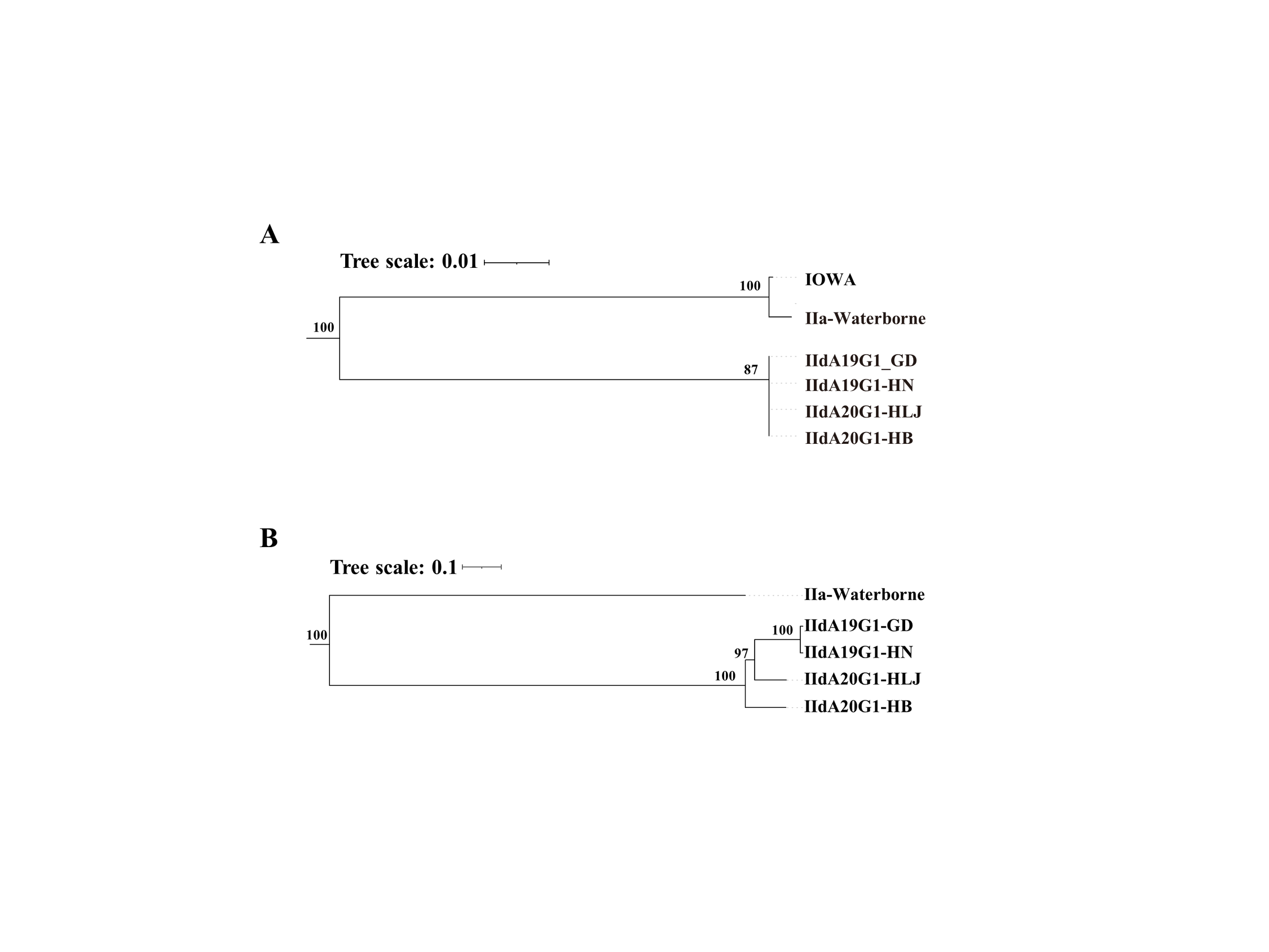

Supplement: S4 Fig — Phylogenetic relationship of the Cryptosporidium parvum IId and IIa isolates based on maximum likelihood analysis of (A) nucleotide sequences of 60 kDa glycoprotein (gp60) gene and (B) genome-wide single nucleotide variants (SNVs) with comparison to the assembled IIdA19G1-GD genome. The numbers on the branches are percent bootstrapping values from 1000 replicates. (TIF) [file pntd.0010714.s004.tif]
