# Supplementary material for: High infectivity and unique genomic sequence characteristics of Cryptosporidium parvum in China
Source: PLoS Negl Trop Dis. 2022 Aug 22;16(8):e0010714. doi: 10.1371/journal.pntd.0010714 (PMC9436107; doi:10.1371/journal.pntd.0010714)
Supplement: S1 Table — (DOCX) [file pntd.0010714.s005.docx]

**S1 Table. Information of five *Cryptosporidium parvum* isolates examined in this study and summary of whole genome sequencing (WGS) data.**

| **Isolate** | **Subtype** | **Host** | **Location** | **Sample name** | **BioProject** | **SRA run** | **Sequence ID** | **Depth** | **Mapping Coverage (%)** | **Number of contigs** | **Assembly length (Mb)** | **N50^a^ (bp)** |
| --- | --- | --- | --- | --- | --- | --- | --- | --- | --- | --- | --- | --- |
| IIa-Waterborne | IIaA17G2R1 | Bovine | Iowa, USA | O18 | PRJNA810562 | SRR18148941 | D20122370 | 381.78 | 99.64 | 106 | 9.05 | 198449 |
| IIdA19G1-HN | IIdA19G1 | Bovine | Henan (HN), central China | O20 |  | SRR18148940 | D1908620 | 97.73 | 99.73 | 203 | 9.10 | 148633 |
| IIdA19G1-GD | IIdA19G1 | Bovine | Guangdong (GD), southeastern China | 22971 |  | SRR18148939 | D1908637 | 94.70 | 99.77 | 196 | 9.10 | 135898 |
| IIdA20G1-HLJ | IIdA20G1 | Bovine | Heilongjiang (HLJ), northeastern China | 11730 |  | SRR18148938 | D1908613 | 100.23 | 99.78 | 137 | 9.12 | 162556 |
| IIdA20G1-HB | IIdA20G1 | Bovine | Hebei (HB), northern China | 12536 |  | SRR18148937 | D1908630 | 115.17 | 99.75 | 164 | 9.11 | 148857 |

^a^ N50, the number of contigs (sorted by length from longest to shortest) whose length when summed covers 50% of the genome assembly.
