# Supplementary material for: High infectivity and unique genomic sequence characteristics of Cryptosporidium parvum in China
Source: PLoS Negl Trop Dis. 2022 Aug 22;16(8):e0010714. doi: 10.1371/journal.pntd.0010714 (PMC9436107; doi:10.1371/journal.pntd.0010714)
Supplement: S2 Table — (DOCX) [file pntd.0010714.s006.docx]

**S2 Table. Primers of nested PCR for confirmation of the major insertion at the 5' end of chromosome 5 in *Cryptosporidium parvum* IId genomes.**

| **Nested PCR** | **Primers** | **Oligonucleotides** | **Products (bp)** |
| --- | --- | --- | --- |
| primary PCR | IId-C5-F1 | 5'-CAC TTG ATT GGC AGA CCA AAT TAA TTC TTG AC-3' | 985 |
|  | IId-C5-R1 | 5'-CAA TTT CTT GGT TAA CTT CCG GAT AG-3' |  |
| secondary PCR | IId-C5-F2 | 5'-CCT GAT AAT TTG ATA CTG GTT TTG CAG C-3' | 748 |
|  | IId-C5-R2 | 5'-CTG TAC ACC CAA ATA ATT CGA ATA CTA G-3' |  |
