# Supplementary material for: High infectivity and unique genomic sequence characteristics of Cryptosporidium parvum in China
Source: PLoS Negl Trop Dis. 2022 Aug 22;16(8):e0010714. doi: 10.1371/journal.pntd.0010714 (PMC9436107; doi:10.1371/journal.pntd.0010714)
Supplement: S3 Table — (DOCX) [file pntd.0010714.s007.docx]

**S3 Table. Common highly polymorphic genes^a^ in *Cryptosporidium parvum* IId genomes with comparison to the genome of IIa-Waterborne.**

| **Gene name in *C. parvum* IOWA** | **Annotation** | **With signal peptide?** | **With transmembrane domain?** | **Length of genes (bp)** | **IIdA19G1-GD** | | **IIdA19G1-HN** | | **IIdA20G1-HLJ** | | **IIdA20G1-HB** | |
| --- | --- | --- | --- | --- | --- | --- | --- | --- | --- | --- | --- | --- |
|  |  |  |  |  | **No. of SNVs** | **Nucleotide identity** | **No. of SNVs** | **Nucleotide identity** | **No. of SNVs** | **Nucleotide identity** | **No. of SNVs** | **Nucleotide identity** |
| cgd1_110 | Signal peptide region containing protein | Yes | Yes | 1221 | 20 | 0.984 | 20 | 0.984 | 18 | 0.985 | 18 | 0.985 |
| cgd1_120 | Signal peptide region containing protein | Yes | No | 1296 | 28 | 0.978 | 24 | 0.981 | 24 | 0.981 | 27 | 0.979 |
| cgd1_130 | Signal peptide region containing protein | Yes | No | 2073 | 24 | 0.988 | 23 | 0.989 | 25 | 0.988 | 24 | 0.988 |
| cgd1_140 | CpLSP gene family | Yes | No | 2892 | 22 | 0.992 | 22 | 0.992 | 21 | 0.993 | 21 | 0.993 |
| cgd1_150 | SKSR gene family | Yes | Yes | 2988 | 21 | 0.993 | 21 | 0.993 | 21 | 0.993 | 21 | 0.993 |
| cgd1_160 | SKSR gene family | Yes | Yes | 2802 | 15 | 0.995 | 15 | 0.995 | 15 | 0.995 | 15 | 0.995 |
| cgd1_400 | MAPK protein kinase | Yes | No | 2187 | 18 | 0.992 | 18 | 0.992 | 18 | 0.992 | 18 | 0.992 |
| cgd1_420 | Putative 20S proteasome beta subunit D2 | No | No | 727 | 4 | 0.994 | 4 | 0.994 | 4 | 0.994 | 4 | 0.994 |
| cgd1_430 | p24-like protein | Yes | Yes | 745 | 8 | 0.989 | 8 | 0.989 | 8 | 0.989 | 8 | 0.989 |
| cgd1_450 | Uncharacterized protein | No | No | 1767 | 22 | 0.988 | 22 | 0.988 | 22 | 0.988 | 22 | 0.988 |
| cgd1_460 | Uncharacterized protein | No | No | 1815 | 38 | 0.979 | 39 | 0.979 | 39 | 0.979 | 39 | 0.979 |
| cgd1_470 | Mucin-like protein | Yes | Yes | 2775 | 20 | 0.993 | 24 | 0.991 | 19 | 0.993 | 19 | 0.993 |
| cgd1_490 | Uncharacterized protein | No | No | 1249 | 26 | 0.979 | 26 | 0.979 | 26 | 0.979 | 26 | 0.979 |
| cgd1_493 | Uncharacterized protein | No | No | 805 | 15 | 0.981 | 15 | 0.981 | 15 | 0.981 | 15 | 0.981 |
| cgd1_500 | Exonuclease V | No | No | 1341 | 16 | 0.988 | 16 | 0.988 | 16 | 0.988 | 16 | 0.988 |
| cgd1_510 | Uncharacterized protein | No | No | 1785 | 24 | 0.987 | 24 | 0.987 | 24 | 0.987 | 24 | 0.987 |
| cgd1_540 | V-type proton ATPase proteolipid subunit | No | Yes | 498 | 5 | 0.990 | 5 | 0.990 | 5 | 0.990 | 5 | 0.990 |
| cgd1_543 | C2H2-type Zinc finger containing protein | No | No | 690 | 10 | 0.985 | 10 | 0.985 | 10 | 0.985 | 10 | 0.985 |
| cgd2_2900 | Uncharacterized protein | Yes | No | 785 | 8 | 0.990 | 8 | 0.990 | 8 | 0.990 | 8 | 0.990 |
| cgd3_1160 | Uncharacterized protein | No | Yes | 1488 | 14 | 0.991 | 15 | 0.990 | 15 | 0.990 | 15 | 0.990 |
| cgd3_1170 | Uncharacterized protein | Yes | No | 903 | 7 | 0.992 | 7 | 0.992 | 7 | 0.992 | 7 | 0.992 |
| cgd3_753 | Uncharacterized protein | No | No | 752 | 4 | 0.995 | 4 | 0.995 | 4 | 0.995 | 4 | 0.995 |
| cgd5_4200 | Transcription initiation factor IIA | No | No | 336 | 2 | 0.994 | 2 | 0.994 | 2 | 0.994 | 2 | 0.994 |
| cgd6_30 | Uncharacterized protein | Yes | No | 822 | 11 | 0.987 | 11 | 0.987 | 11 | 0.987 | 11 | 0.987 |
| cgd6_40 | Mucin-like protein | Yes | Yes | 579 | 12 | 0.979 | 13 | 0.978 | 13 | 0.978 | 12 | 0.979 |
| cgd6_1080 | GP60 | Yes | Yes | 981 | 8 | 0.992 | 9 | 0.991 | 9 | 0.991 | 8 | 0.992 |
| cgd6_3940 | Uncharacterized protein | Yes | No | 1107 | 11 | 0.990 | 9 | 0.992 | 9 | 0.992 | 10 | 0.991 |
| cgd6_5460 | FLGN gene family | Yes | No | 1290 | 17 | 0.987 | 17 | 0.987 | 17 | 0.987 | 17 | 0.987 |
| cgd6_5470 | Putative signal peptide-containing protein | No | Yes | 657 | 4 | 0.994 | 5 | 0.992 | 5 | 0.992 | 5 | 0.992 |
| cgd7_4180 | Clathrin adaptor complex small chain/Longin-like domain containing protein | No | No | 589 | 4 | 0.993 | 4 | 0.993 | 4 | 0.993 | 4 | 0.993 |
| cgd7_4450 | Elongation factor EF1-gamma (Glutathione S-transferase family) | No | No | 1149 | 7 | 0.994 | 7 | 0.994 | 7 | 0.994 | 7 | 0.994 |

^a^ Common highly polymorphic genes were identified using the mean + 3 standard deviation values of single nucleotide variants (SNVs).
