# Supplementary material for: High infectivity and unique genomic sequence characteristics of Cryptosporidium parvum in China
Source: PLoS Negl Trop Dis. 2022 Aug 22;16(8):e0010714. doi: 10.1371/journal.pntd.0010714 (PMC9436107; doi:10.1371/journal.pntd.0010714)
Supplement: S4 Table — (DOCX) [file pntd.0010714.s008.docx]

**S4 Table. Summary of single nucleotide variants (SNVs) in IId genomes sequenced in this study compared with the genome of IIdA19G1-GD.**

| **Isolate** | **No. of SNVs** | **No. of SNVs in genes** | **No. of non-synonymous SNVs** |
| --- | --- | --- | --- |
| IIdA19G1-HN | 77 | 55 | 28 |
| IIdA20G1-HLJ | 1158 | 780 | 409 |
| IIdA20G1-HB | 1452 | 943 | 509 |
