# Supplementary material for: High infectivity and unique genomic sequence characteristics of Cryptosporidium parvum in China
Source: PLoS Negl Trop Dis. 2022 Aug 22;16(8):e0010714. doi: 10.1371/journal.pntd.0010714 (PMC9436107; doi:10.1371/journal.pntd.0010714)
Supplement: S5 Table — (DOCX) [file pntd.0010714.s009.docx]

**S5 Table. Highly polymorphic genes^a^ between *Cryptosporidium parvum* IIdA19G1-GD and the other three IId isolates, IIdA19G1-HN, IIdA20G1-HLJ and IIdA20G1-HB.**

| **Gene name in**  ***C. parvum* IOWA** | **Annotation** | **With signal peptide?** | **With transmembrane domain?** | **Length of genes (bp)** | **IIdA19G1-HN** | | **IIdA20G1-HLJ** | | **IIdA20G1-HB** | |
| --- | --- | --- | --- | --- | --- | --- | --- | --- | --- | --- |
|  |  |  |  |  | **No. of SNVs** | **Nucleotide identity** | **No. of SNVs** | **Nucleotide identity** | **No. of SNVs** | **Nucleotide identity** |
| cgd1_3850 | SKSR gene family | Yes | Yes | 2775 | - | - | 10 | 0.996 | - | - |
| cgd1_3860 | dUTPase-like protein | No | Yes | 699 | - | - | 4 | 0.994 | - | - |
| cgd2_3270 | Phosphoglucomutase | No | No | 2013 | 10 | 0.995 | - | - | - | - |
| cgd2_3860 | BBOX Zinc finger domain containing protein | No | No | 1575 | - | - | - | - | 3 | 0.998 |
| cgd2_420 | Mucin-like protein | Yes | No | 594 | - | - | 2 | 0.997 | - | - |
| cgd2_750 | WD40 repeat containing protein | No | No | 1269 | - | - | 4 | 0.997 | 4 | 0.997 |
| cgd3_120 | Chromosome segregation protein Spc25 | No | No | 687 | - | - | - | - | 2 | 0.997 |
| cgd3_3600 | BTB/POZ domain/Kelch motif/Galactose oxidase central domain containing protein | No | No | 1581 | - | - | 4 | 0.997 | - | - |
| cgd3_3620 | Uncharacterized protein with Armadillo-type fold | No | Yes | 4629 | - | - | - | - | 9 | 0.998 |
| cgd3_360 | Uncharacterized protein | No | No | 393 | - | - | 2 | 0.995 | 2 | 0.995 |
| cgd3_60 | Uncharacterized protein | No | No | 750 | - | - | - | - | 2 | 0.997 |
| cgd4_10 | Uncharacterized protein | Yes | No | 483 | - | - | 4 | 0.992 | - | - |
| cgd4_20 | Uncharacterized protein | Yes | No | 1008 | - | - | 15 | 0.985 | - | - |
| CPATCC_001474^b^ | Uncharacterized protein | No | Yes | 288 | - | - | 3 | 0.990 | - | - |
| cgd4_30 | Uncharacterized protein | No | No | 1341 | - | - | 6 | 0.996 | - | - |
| cgd5_4610 | MEDLE gene family | No | No | 716 | - | - | 4 | 0.994 | 3 | 0.996 |
| cgd_6g5^c^ | Uncharacterized protein | No | No | 714 | - | - | 3 | 0.996 | - | - |
| cgd6_110 | Malectin | Yes | Yes | 858 | - | - | 2 | 0.998 | - | - |
| cgd6_20 | Inosine-5-monophosphate dehydrogenase | No | No | 1203 | - | - | 16 | 0.987 | - | - |
| cgd6_4740 | Uncharacterized protein | No | Yes | 834 | - | - | 3 | 0.996 | 3 | 0.996 |
| cgd6_4750 | Splicing factor 3B subunit 1 | No | No | 3096 | - | - | 19 | 0.994 | 19 | 0.994 |
| cgd6_4770 | Torus/RNA recognition motif domain containing protein | No | No | 1029 | - | - | 3 | 0.997 | 3 | 0.997 |
| cgd6_4780 | Uncharacterized protein | No | Yes | 1221 | - | - | 4 | 0.997 | 5 | 0.996 |
| cgd6_4790 | Uncharacterized protein with B-box-type zinc finger | No | No | 3477 | - | - | 9 | 0.997 | 8 | 0.998 |
| cgd6_4800 | P-loop containing nucleoside triphosphate hydrolase | No | No | 1320 | - | - | 5 | 0.996 | 5 | 0.996 |
| cgd6_4830 | DEAD/DEAH box helicase | No | No | 1632 | - | - | 4 | 0.998 | 4 | 0.998 |
| cgd6_4840 | Serine protease, subtilase family | Yes | No | 4051 | - | - | 7 | 0.998 | 7 | 0.998 |
| cgd6_4850 | Pre-mRNA-splicing factor 19 with U box domain | No | No | 1656 | - | - | 5 | 0.997 | 5 | 0.997 |
| cgd6_4890 | Chloroquine resistance transporter | No | Yes | 1341 | - | - | - | - | 3 | 0.998 |
| cgd6_4910 | Zinc finger C3H1-type domain containing protein | No | No | 1764 | - | - | - | - | 4 | 0.998 |
| cgd6_4920 | Uncharacterized protein | No | Yes | 1722 | - | - | - | - | 4 | 0.998 |
| cgd5_4510 | Uncharacterized protein | Yes | Yes | 987 | - | - | - | - | 2 | 0.998 |
| cgd7_4500 | Uncharacterized Secreted Protein | Yes | No | 2496 | - | - | - | - | 35 | 0.986 |
| cgd7_4910 | Zinc finger%2C RING/FYVE/PHD-type domain containg protein | No | Yes | 936 | - | - | - | - | 2 | 0.998 |
| CPATCC_003712^d^ | Secreted glycosyltransferase | No | Yes | 966 | - | - | - | - | 3 | 0.997 |
| cgd7_5050 | NIMA-related kinase 5 | No | No | 4188 | - | - | - | - | 10 | 0.998 |
| cgd7_5070 | Uncharacterized protein | No | No | 614 | - | - | 4 | 0.993 | - | - |
| cgd7_5320 | Cwf5-like/ZnR domain containing protein (CCCH and RRM domains missing) | No | No | 561 | - | - | - | - | 2 | 0.996 |
| cgd8_100 | Uncharacterized protein | No | No | 3750 | 2 | 0.999 | - | - | - | - |
| cgd8_1160 | Mucin-like protein | Yes | Yes | 1560 | - | - | - | - | 4 | 0.997 |
| cgd8_2400 | Uncharacterized protein | No | No | 750 | - | - | 2 | 0.997 | 2 | 0.997 |
| cgd8_2463 | Uncharacterized protein | No | No | 1785 | - | - | - | - | 4 | 0.998 |
| cgd8_3703 | Nucleolar protein 12 | No | No | 460 | - | - | 2 | 0.996 | - | - |
| cgd8_4210 | Uncharacterized protein | No | No | 900 | - | - | - | - | 2 | 0.998 |
| cgd8_4220 | High mobility group box domain containing protein | No | No | 929 | - | - | - | - | 2 | 0.998 |
| cgd8_660 | Uncharacterized protein | No | No | 5922 | 3 | 0.999 | - | - | - | - |

^a^ Common highly polymorphic genes were identified using the mean + 3 standard deviation values of single nucleotide variants (SNVs)

^b d^ Orthologs found in genome of IOWA-ATCC.

^c^ Ortholog found in unpublished genome of IOWA.
